# Dapagliflozin-Induced Dermatological Condition: Sweet’s Syndrome

**DOI:** 10.7759/cureus.110647

**Published:** 2026-06-11

**Authors:** Nourah Alazemi, Adnan Ahmad, Alsadat Mosbeh, Abeer Albazali

**Affiliations:** 1 Dermatology, Kuwait Institute for Medical Specializations, Ministry of Health, Kuwait City, KWT; 2 Dermatology, Farwaniya Hospital, Kuwait City, KWT; 3 Dermatology/Dermatopathology, Faculty of Medicine, Al-Azhar University, Cairo, EGY

**Keywords:** corticosteroids, febrile neutrophilic dermatosis, neutrophilia, poor glycemic control, sweet’s syndrome

## Abstract

Sweet's syndrome, or acute febrile neutrophilic dermatosis, is an uncommon inflammatory condition characterized by the sudden onset of fever, neutrophilia, and painful erythematous skin lesions. We present the case of a 54-year-old female who was started on anti-glycemic medication for glycemic control and developed tender erythematous plaques and nodules over the upper limbs. Comprehensive evaluation excluded infectious, autoimmune, endocrine, and neoplastic causes. Skin biopsy demonstrated a dense neutrophilic infiltrate without evidence of vasculitis, supporting the diagnosis of Sweet’s syndrome. The patient was managed with systemic corticosteroids, resulting in clinical improvement. This case emphasizes the importance of recognizing Sweet’s syndrome in the context of drug-induced etiology, as well as the role of timely dermatologic assessment and metabolic stabilization in achieving favorable outcomes.

## Introduction

Sweet’s syndrome, also known as acute febrile neutrophilic, is a rare inflammatory condition marked by the rapid onset of painful erythematous plaques or nodules, typically accompanied by fever and peripheral neutrophilia. The disorder is broadly classified into three subtypes: classical (idiopathic or post-infectious), malignancy-associated, and drug-induced [1,2]. Histologically, it is characterized by sub-epidermal edema and a dense neutrophilic infiltrate within the upper dermis without evidence of true leukocytoclastic vasculitis, a key diagnostic feature [1,2].

Classical Sweet’s syndrome represents the majority of cases and is frequently preceded by an upper respiratory or gastrointestinal infection. Its pathogenesis is believed to involve an abnormal cytokine-driven neutrophilic response triggered by infection, immune dysregulation, or systemic inflammation, resulting in dermal neutrophilic infiltration in the absence of true leukocytoclastic vasculitis - a key histopathological hallmark [2]. Owing to its significant clinical overlap with conditions, such as cellulitis, erythema nodosum, vasculitis, and other neutrophilic dermatoses, early recognition is essential to prevent unnecessary antibiotic use and to allow timely initiation of systemic corticosteroids, which typically produce a rapid and marked clinical improvement [3].

## Case presentation

A 54-year-old Indian female with a history of type 2 diabetes mellitus was started on dapagliflozin (5 mg) for glycemic control. Within three days of starting the medication, she developed painful skin lesions over both upper limbs. These lesions were described as tender, erythematous plaques and nodules predominantly involving the extensor aspects of the forearms and arms. The lesions were associated with significant discomfort but no pruritus, and no satellite lesions on the dorsal aspects of the hands. The patient reported a history of fever (38.3°C), which started several days after the onset of the cutaneous lesions, but denied other systemic symptoms, such as respiratory, gastrointestinal, or urinary complaints. She denied any history of trauma, insect bites, or similar prior episodes. She had no surgical history, no recent travel, no toxic exposures, no herbal supplements, and no drug allergies. She reported no recent direct contact with animals.

**Figure 1:**
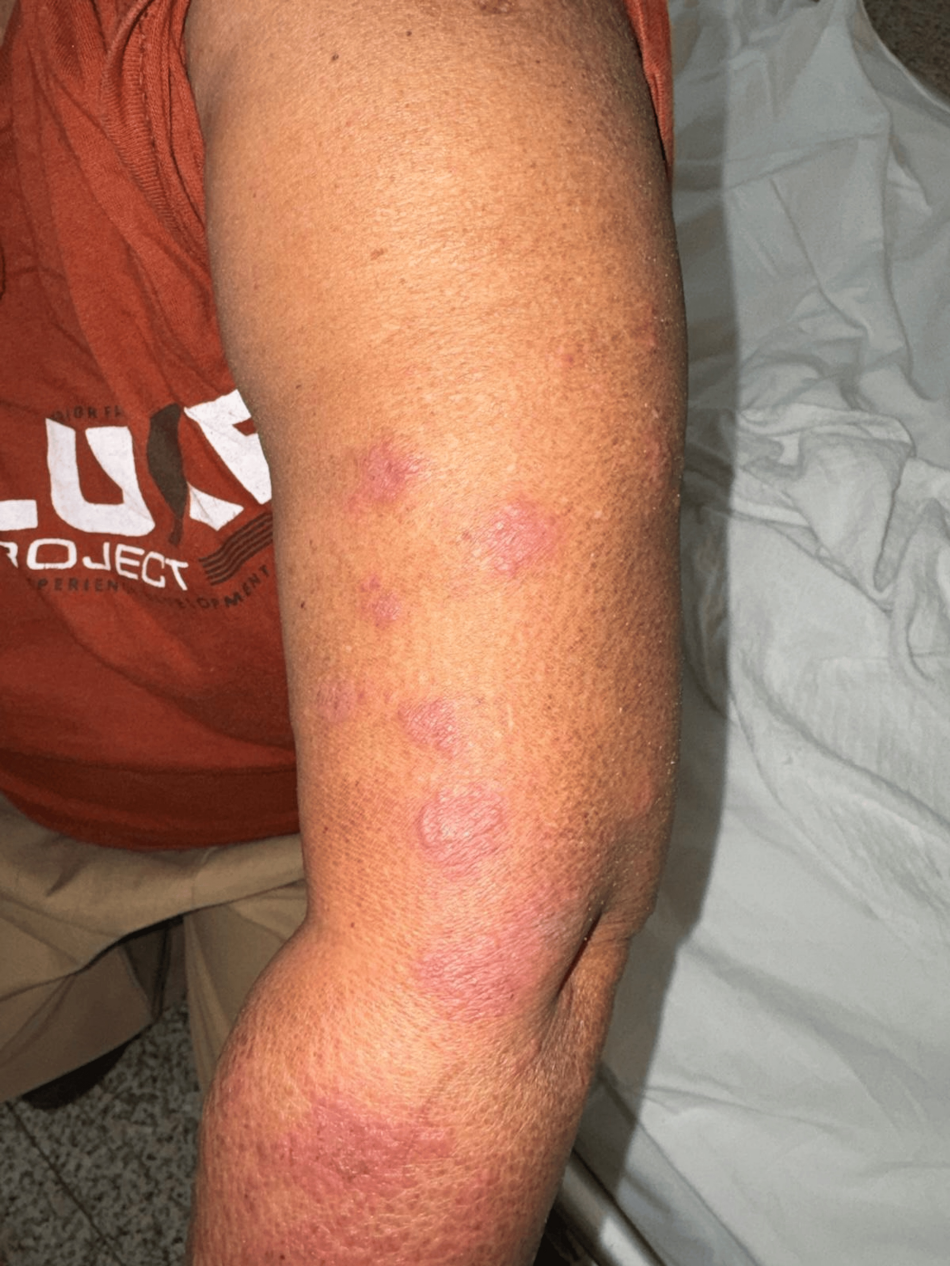
Multiple tender, erythematous nodules with minute pustules on the upper limbs at presentation

Examination revealed multiple tender, indurated, erythematous nodules of varying size distributed across both upper limbs (more exuberant on the right arm) (Figure 1), abdomen, and face. Several lesions exhibited minute pustules. No mucosal involvement was present. Cardiopulmonary, abdominal, and neurological examinations were otherwise unremarkable.

Initial laboratory studies showed a high leukocyte count with neutrophil predominance, elevated C-reactive protein (CRP), preserved renal function, and normal electrolytes and liver enzymes (Table 1). Urinalysis was unremarkable. The respiratory viral PCR panel was negative. The chest radiograph was unremarkable.

**Table 1:** Laboratory findings on admission and evolution. ALP, alkaline phosphatase; ALT, alanine aminotransferase; aPTT, activated partial thromboplastin time; AST, aspartate aminotransferase; CRP, C-reactive protein; ESR, erythrocyte sedimentation rate; GGT, gamma-glutamyl transferase; INR, international normalized ratio; LDH, lactate dehydrogenase; MCH, mean corpuscular hemoglobin; MCHC, mean corpuscular hemoglobin concentration; MCV, mean corpuscular volume; RBC, red blood cell; WBC, white blood cell; β-hCG, beta human chorionic gonadotropin

Parameter	Admission	Evolution	Reference range
Hemoglobin	12.2 g/dL	12.5 g/dL	12-16 g/dL
Hematocrit	35.6 %	32.6 %	36-46%
RBC count	4.39 ×10⁶/µL	3.85 ×10⁶/µL	4.0-5.2 × 10⁶/µL
MCV	83.8 fL	-	83-103 fL
MCH	30.3 pg	-	28-34 pg
MCHC	36.1 g/dL	-	32-36 g/dL
RDW	11.9 %	-	-
WBC count	11.3 ×10³/µL	7.8 ×10³/µL	4.8-10.8 × 10³/µL
Neutrophils	88.8 % (7.58 ×10³/µL)	63.2 %	1.8-7.7 × 10³/µL
Lymphocytes	8.4 % (0.89 ×10³/µL)	-	1.0-4.8 × 10³/µL
Monocytes	8.9 % (0.94 ×10³/µL)	-	0.12-0.80 × 10³/µL
Eosinophils	0.1 %	-	0.00-0.49%
Basophils	0.2 %	-	0.0-0.1%
Platelets	235 ×10³/µL	-	150-350 × 10³/µL
ESR	105 mm	-	0-12 mm
CRP	210.2 mg/L	16.0 mg/L	<3 mg/L
Procalcitonin	0.10 ng/mL	-	<0.1 / <0.5 ng/mL
Urea	23 mg/dL	-	15-39 mg/dL
Creatinine	0.48 mg/dL	0.56 mg/dL	0.55-1.02 mg/dL
Sodium	136 mEq/L	140 mEq/L	135-146 mEq/L
Potassium	4.00 mEq/L	3.70 mEq/L	3.5-5.1 mEq/L
Chloride	99 mEq/L	-	95-105 mEq/L
Calcium	8.9 mg/dL	-	8.3-10.6 mg/dL
Phosphorus	3.5 mg/dL	-	2.5-4.9 mg/dL
Magnesium	2.47 mg/dL	-	1.6-2.6 mg/dL
AST	22 U/L	15 U/L	12-40 U/L
ALT	10 U/L	13 U/L	7-40 U/L
GGT	12 U/L	-	0-38 U/L
ALP	39 U/L	-	46-116 U/L
LDH	350 U/L	195 U/L	120-246 U/L
Albumin	4.7 g/dL	-	3.4-5.0 g/dL
Total protein	8.1 g/dL	-	5.7-8.2 g/dL
Ferritin	368 ng/mL	-	10-291 ng/mL
Transferrin	159 mg/dL	-	250-380 mg/dL
β-hCG	<2 mIU/mL	-	<5 (non-pregnant)
INR	1	-	Normal
aPTT	28.3 s	-	Normal

**Figure 2:**
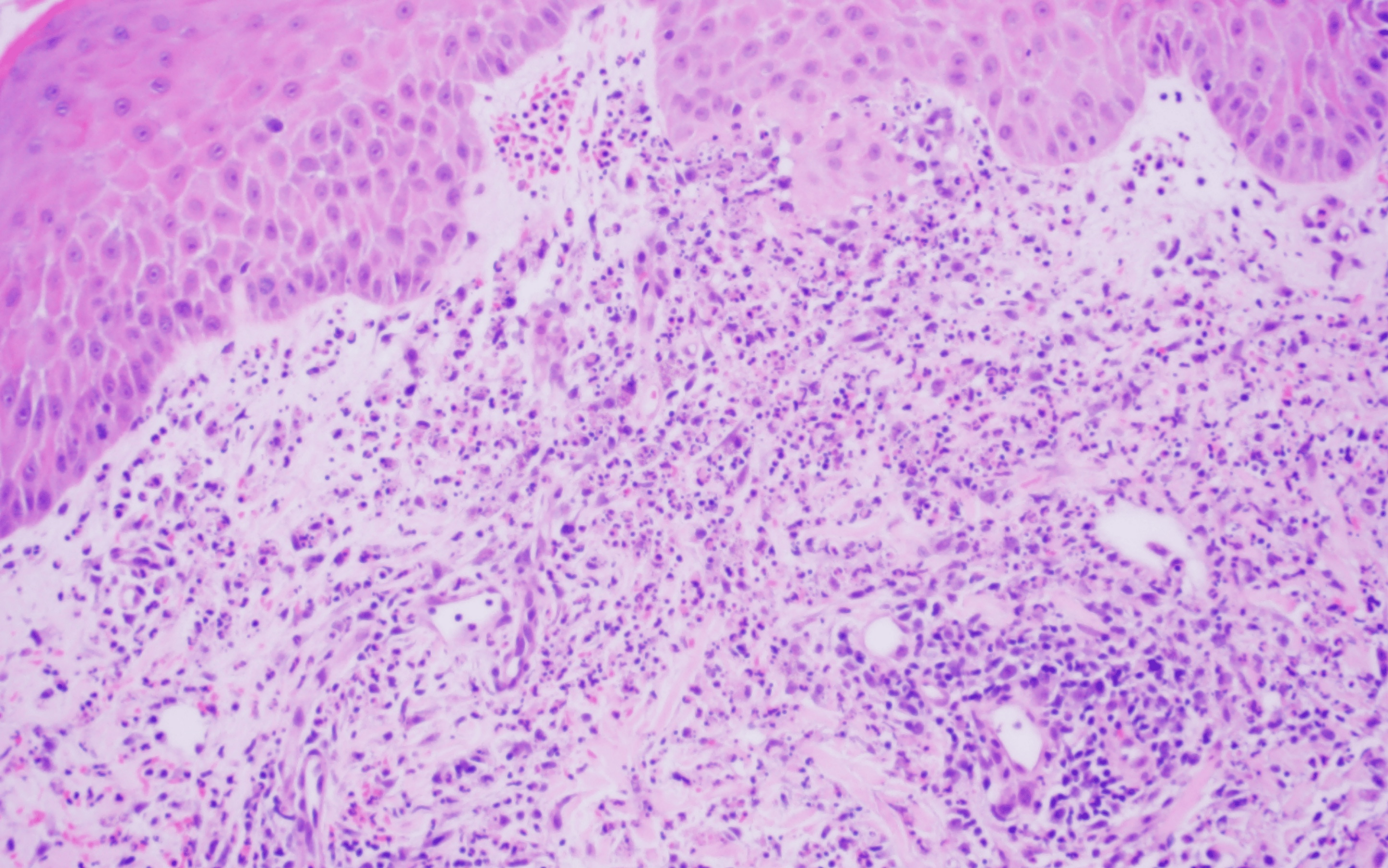
High-power (×20) examination of skin biopsy Demonstrates papillary sub-epidermal edema with dermal infiltration (mainly neutrophils) with extravasated red blood cells and leukocytoclasia

The patient was admitted to the hospital for further evaluation and management. Dermatology consultation raised suspicion for Sweet’s syndrome. Dapagliflozin was discontinued, as this medication was thought to be triggering the eruption. Systemic corticosteroid therapy with prednisolone 1 mg/kg/day was initiated immediately after performing the skin biopsy of the right arm lesion. Fever improved within 48-72 hours, and the skin lesions progressively resolved over the ensuing one to two weeks. The histopathology report confirmed Sweet’s syndrome, demonstrating a dense neutrophilic infiltrate in the upper dermis with papillary dermal edema, with the absence of vasculitis (Figure 2). No microorganisms were identified.

A comprehensive etiological evaluation was undertaken. Screening for HIV, HBV, and HCV was negative. Serology showed past exposure to rubella, varicella-zoster virus, cytomegalovirus, toxoplasmosis, and Epstein-Barr virus. Syphilis screening was negative. Blood and urine cultures were ultimately negative. CT of the thorax, abdomen, and pelvis detected no occult neoplasia or systemic involvement. Endocrine evaluation revealed normal thyroid function. No evidence of extra-cutaneous Sweet’s syndrome was identified throughout the systemic evaluation.

Laboratory follow-up performed at the same 48-hour mark also demonstrated a clear analytical improvement, with a marked decline in white blood cells (7.8 x 10³ mL) and CRP (16 mg/L). By the third day of treatment, the patient regained independent mobility. The cutaneous nodules gradually became darker and flatter, with no development of new lesions.

The patient was discharged on a structured prednisolone taper of 10 mg per week and followed up in the outpatient Dermatology Department. She remained under clinical follow-up with periodic reassessment, with no clinical relapse or laboratory abnormalities to date, with complete cutaneous remission and no recurrence, which is relevant given that relapse has been reported in a significant proportion of patients in published series [3,4].

## Discussion

Diagnosis of Sweet's syndrome is established when both major criteria - typical painful erythematous plaques and characteristic neutrophilic dermal infiltrate without vasculitis - are present along with at least two minor criteria, such as fever, preceding infection or inflammatory trigger, elevated inflammatory markers or neutrophilia, and rapid response to corticosteroids. Although Sweet’s syndrome is classically associated with leukocytosis, it may also present with relative neutrophilia and markedly elevated inflammatory markers even in the absence of overt leukocytosis, as documented in published series [1-4].

The extensive etiological work-up was essential to exclude other subtypes. There was no evidence of hematological disease, solid malignancy, or active infection.

Corticosteroids remain the treatment of choice; most patients respond within 48 to 72 hours. Alternative agents, such as colchicine, dapsone, or potassium iodide, may be reserved for recurrent or refractory disease [5]. The risk of relapse is reported in up to one-third of patients, especially in the presence of persistent triggers or insufficient tapering [6]. This patient demonstrated an excellent response with no recurrence to date. In some patients with classical Sweet's syndrome, the symptoms and lesions of Sweet's syndrome eventually resolved without any therapeutic intervention. However, the lesions may persist for weeks to months [7,8]. Similarly, spontaneous improvement and subsequent resolution of the syndrome typically follow discontinuation of the associated medication in patients with drug-induced Sweet's syndrome [9,10].

Although the precise mechanism remains unclear, dapagliflozin is thought to trigger Sweet syndrome through an idiosyncratic immune-mediated reaction leading to cytokine dysregulation and neutrophil hyperactivation. Current evidence suggests that drug-induced Sweet syndrome is driven by increased production of pro-inflammatory cytokines, including interleukin (IL)-1, IL-6, IL-8, tumor necrosis factor-alpha (TNF-α), and granulocyte colony-stimulating factor (G-CSF), which promote neutrophil recruitment and infiltration of the dermis. Recent reviews of sodium-glucose cotransporter 2 (SGLT2) inhibitor-associated cutaneous adverse reactions have identified Sweet syndrome as a rare but recognized complication, supporting a role for immune dysregulation rather than the drug's glucose-lowering mechanism. The temporal relationship between dapagliflozin exposure, symptom onset, and clinical improvement following drug withdrawal further supports a causal association [10]. 

The temporal use of dapagliflozin and appearance of the hand lesions, along with the histology, favored drug-induced Sweet syndrome due to dapagliflozin as the cause of the plaques. 

## Conclusions

Sweet's syndrome is characterized by four cardinal features: fever, leukocytosis, tender erythematous plaques, and a dense dermal neutrophilic infiltrate. Histopathologically, the condition demonstrates a nodular neutrophilic infiltrate accompanied by papillary dermal edema and leukocytoclastic debris. In cases of drug-induced Sweet syndrome, a clear temporal association exists between medication exposure and symptom onset, with improvement typically occurring after withdrawal of the offending agent or initiation of systemic corticosteroid therapy. Importantly, dapagliflozin, a member of the newer gliflozin class of medications used in the treatment of type 2 diabetes mellitus, should be recognized as a potential trigger for drug-induced Sweet's syndrome.

## References

[1] Joshi TP, Friske SK, Hsiou DA, Duvic M (2022). New practical aspects of Sweet syndrome. Am J Clin Dermatol.

[2] SW RD (1964). An acute febrile neutrophilic dermatosis. Br J Dermatol.

[3] Villarreal-Villarreal CD, Ocampo-Candiani J, Villarreal-Martínez A (2016). Sweet syndrome: a review and update. Actas Dermosifiliogr.

[4] Jung EH, Park JH, Hwan Kim K (2022). Characteristics of Sweet syndrome in patients with or without malignancy. Ann Hematol.

[5] Walker D, Cohen P (1996). Trimethoprim-sulfamethoxazole-associated acute febrile neutrophilic dermatosis: case report and review of drug-induced Sweet's syndrome. J Am Acad Dermatol.

[6] Xie Y, Li Q, Zhou J, Qin X, Zhang Z, Bai R (2025). Drug-induced Sweet's syndrome: pharmacovigilance insights from FAERS with a cross-database consistency assessment in VigiBase via LASSO and multivariable logistic regression. Front Immunol.

[7] Cohen PR, Kurzrock R (2000). Sweet's syndrome: a neutrophilic dermatosis classically associated with acute onset and fever. Clin Dermatol.

[8] Walker DC, Cohen PR (1996). Trimethoprim-sulfamethoxazole-associated acute febrile neutrophilic dermatosis: case report and review of drug-induced Sweet's syndrome. J Am Acad Dermatol.

[9] Tavadia SM, Smith G, Herd RM, Zuk RJ (1999). Sweet's syndrome associated with oral squamous cell carcinoma and exhibiting the Koebner phenomenon. Br J Dermatol.

[10] Mederle AL, Dumitrescu P, Borza C, Kundnani NR (2024). Cutaneous adverse drug reactions associated with SGLT2 inhibitors. J Clin Med.

